# Hospital Construction

**Published:** 1916-05-13

**Authors:** R. Langton Cole

**Affiliations:** 23 Throgmorton Street, E.C.


*To the Editor of* The Hospital.

### Hospital Construction.

*To the Editor of* The Hospital.

Sir,—May I suggest that in your articles on this subject the date of erection of the buildings described should always be referred to? In the case of foreign and colonial hospitals (as Toronto), especially, it helps one to know whether the plan represents recent practice (it does—Ed. The Hospital) or an earlier type, and to those who keep your articles the date is often of interest in other ways.—Yours, etc.,

23 Throgmorton Street, E.C., R. Langton Cole.

May 5, 1916.

